# Comparison of obturation quality in natural and replica teeth root-filled using different sealers and techniques

**DOI:** 10.1007/s00784-023-04884-9

**Published:** 2023-02-04

**Authors:** Chuta Kooanantkul, Richard M Shelton, Josette Camilleri

**Affiliations:** School of Dentistry, Institute of Clinical Sciences, College of Medical and Dental Sciences, University of Birmingham, 5, Mill Pool Way, Edgbaston, Birmingham, B5 7EG UK

**Keywords:** Root canal filling quality, Microcomputed tomography, Calcium silicate–based sealer, Obturation technique, 3D printing

## Abstract

**Objectives:**

This study aims to assess the obturation efficacy of sealers placed with different techniques using microcomputed tomography (µCT) and assess the influence of µCT testing parameters on the obturation data obtained.

**Materials and methods:**

Incisors and mesial roots of lower molars with standardized root length were scanned using µCT, and one tooth of each type was 3D printed in acrylic. Two obturation techniques (warm vertical and single cone) and 4 sealer types (AH Plus, BioRoot RCS, Totalfill BC, and Bio-C Sealers) were assessed following storage in Hank’s balanced salt solution for 3 and 6 months by assessing gap and void volume percentages on both natural and replica incisor and molar roots. The storage solution was analysed to assess calcium ion leaching. The influence of temperature, tooth positioning, and moisture content of the teeth while µCT scanning was also investigated.

**Results:**

The obturation quality in the incisor group was the same using both natural teeth and replicas (*p* > 0.05). No changes in void volume were identified when comparing the same sealer using different obturation techniques. The premixed sealers used in single-cone obturation exhibited high void volume in the 3D printed replicas in the long term. The temperature, positioning, and moisture content of the teeth did not affect the outcome of µCT testing.

**Conclusions:**

BioRoot RCS, Totalfill BC, and Bio-C Sealers are suitable for obturation of both complex and simple root canal systems using different obturation techniques with BioRoot RCS exhibiting the highest calcium ion release. 3D printed acrylic teeth can be used to assess the obturation quality in uncomplicated root canal systems. µCT parameters had no significant effect on the µCT measurement.

**Clinical relevance:**

The single-cone obturation technique with hydraulic sealer is a simple technique that can be used for obturation of all root canal systems.

## Introduction

Root canal obturation is necessary to prevent reinfection of the root canal space in the long term. Warm vertically compacted and laterally condensed gutta-percha techniques have been used to hermetically fill the root canal using a combination of gutta-percha and root canal sealant. AH Plus (Dentsply Maillefer, Tulsa, OK, USA) is most commonly used in both techniques due to its low shrinkage (1.76%), low solubility compared with other resin-based sealers, and low film thickness complying with the ISO requirement for root canal sealing materials [1]. However, AH Plus does not fulfil Grossman’s ideal root canal sealer properties [2] as it has no bactericidal effect and shrinks when set [3].

More recently, the single cone/sealer-based technique has become more popular among clinicians due to its simplicity and not requiring specific armamentarium [4]. This technique is used with hydraulic calcium silicate cements (HCSCs) as these sealers have shown antimicrobial characteristics [5–8] and generation of an appropriate seal [3], thus indicating suitability for sealer-based techniques. There are a number of hydraulic cement sealers available clinically which have similar chemistries but different modes of presentation. These sealants are all primarily composed of tricalcium silicate with a radiopacifier and additives to enhance the material properties [9]. These sealers exhibit higher release of calcium ions and demonstrate higher flow rates, higher pH, and lower cytotoxicity than AH Plus [10–12]. Chemical and micromechanical bonds have also been identified with the dentine [1, 13]. The hydrophilicity of the hydraulic sealers has led to some concerns regarding adhesion to the gutta-percha cones. A bioceramic coated gutta-percha (GP) cone (BC cone) was introduced (Brassler, Georgia, USA) to address this issue where the GP was coated and impregnated with calcium silicate nanoparticles aiming to cause interaction with a hydraulic calcium silicate sealer and form an actual gap-free seal (monoblock system). To date, relatively few studies have evaluated this type of GP in terms of filling quality and ion leaching.

Although no link has been definitively demonstrated between root canal obturation quality and clinical success rates, sealing ability has always been used to assess and compare obturation techniques as a surrogate marker. Dyes, glucose, protein, or bacterial penetration from coronal to apical through the obturated root canal and the time taken has several limitations in assessment of sealing ability. Dyes and bacterial penetration vary according to the type of dye and bacteria species [14]. Another method for determining obturation quality is the use of microcomputed tomography (µCT) to assess percentage of gaps and voids (%*V*_*gv*_). µCT can be used to scan and reconstruct the sample in 3-dimesions at µm resolution in a non-invasive manner without causing sample damage [15–17]. It also shows the volume, location, and size of gaps (space between the material and root) and voids (space inside the material). Comparison of data is still challenging since the natural teeth used for these measurements have variable anatomy and shapes. Such variations could be overcome by using standardised 3D printed teeth with the morphology copied from natural teeth.

The objectives of this study were to assess the obturation quality of sealers used with different techniques and the validity of using 3D printed teeth for assessment, determined using µCT. Furthermore, the influence of varying the µCT testing parameters on the data generated were also examined.

## Materials and methods

The following sealers were used with two obturation methods and tested in human teeth and in replicas.

- AH Plus (Dentsply DeTrey, Konstanz, Germany)
- BioRoot RCS (Septodont, Saint-Maur-des-Fosses, France)
- Totalfill BC Sealer (FKG, Chaux de Fonds, Switzerland)
- Bio-C Sealer (Angelus, Londrina, Brazil)

### Sealer characterisation

All sealers were characterised to assess the material microstructure and elemental composition using scanning electron microscopy (SEM; EVO MA10, Carl Zeiss, Cambridge, UK) and energy-dispersive X-ray spectroscopy (EDS; EVO MA10, Carl Zeiss, Cambridge, UK). Sealers were injected into circular rubber moulds 10 mm internal diameter and 2 mm high and placed on a glass mixing slab kept at 37 °C and 100% humidity until the surface was resistant to indentation.

After setting, each sealer was removed from the rubber mould and embedded in epoxy resin (EpoFix, Struers, Ballerup, Denmark). Resin blocks were placed in a fume cupboard at room temperature for 1 day to allow complete polymerisation. After setting, the sample surfaces were ground using 220, 500, and 1200 grit diamond discs (MD-Piano, Struers ApS, Denmark) with water coolant for 1 min each, using an automatic grinding and polishing machine (Phoenix Beta, Buehler, Lake Bluff, IL, USA). Polishing was completed with cloth discs (MD Largo, Dac, Nap, Struers Aps, Ballerup, Denmark) using 9, 3, and 1 µm diamond impregnated polishing liquids (DiaPro, Struers ApS, Ballerup, Denmark) each for 3 min. All specimens were mounted on aluminium stubs with double-sided carbon tape. Each sample was sputter-coated with gold (EMITECH K550X; Carl Zeiss, UK; needle value adjustment was set to give 0.1 mbar of argon gas at a nominal 0.3 bar, deposition current of 25 mA and 2 min deposition time) before examination using SEM and EDS for elemental analysis (working distance 8.5 mm, I Probe 1,000 pA and accelerating voltage 20 kV). The type and weight fraction of the elements present were recorded from EDS using an area analysis.

### Preparation of teeth and resin replicas

The use of human teeth for research was approved by the Research and Innovation Department, Birmingham Community Healthcare Trust (14/EM/1128). Teeth with fully formed apices and root curvatures less than 30° were included in the study, whilst teeth with root caries or restorations or root fractures were excluded. Remnants of periodontal ligament (PDL) and bone were gently removed using a Gracey curette (SG11/12, Hu-Friedy, Chicago, IL, USA). The crowns were removed using a diamond bur, and the roots of the upper incisors were standardized to 16 mm in length, while the mesio-buccal roots were sectioned from lower molars and standardized to 12 mm in length.

The root canal therapy was all undertaken by the same operator, a specialist endodontist. The root canals were prepared using ProTaper Gold (Dentsply Maillefer, Ballaigues, Switzerland) using SX, S1, S2, F1, and F2 series for the molar roots and to F3 for the incisors and copious irrigation with 5.25% sodium hypochlorite (NaOCl; Chloraxid, Cerkamed, Stalowa, Wola, Poland) between each instrumentation. After completing mechanical instrumentation, 3 mL 17% ethylenediamine tetraacetic acid (EDTA; Cerkamed, Stalowa, Wola, Poland) was used to rinse the canal for 1 min, followed by 3 mL 5.25% NaOCl for 1 min.

**Fig. 1:**
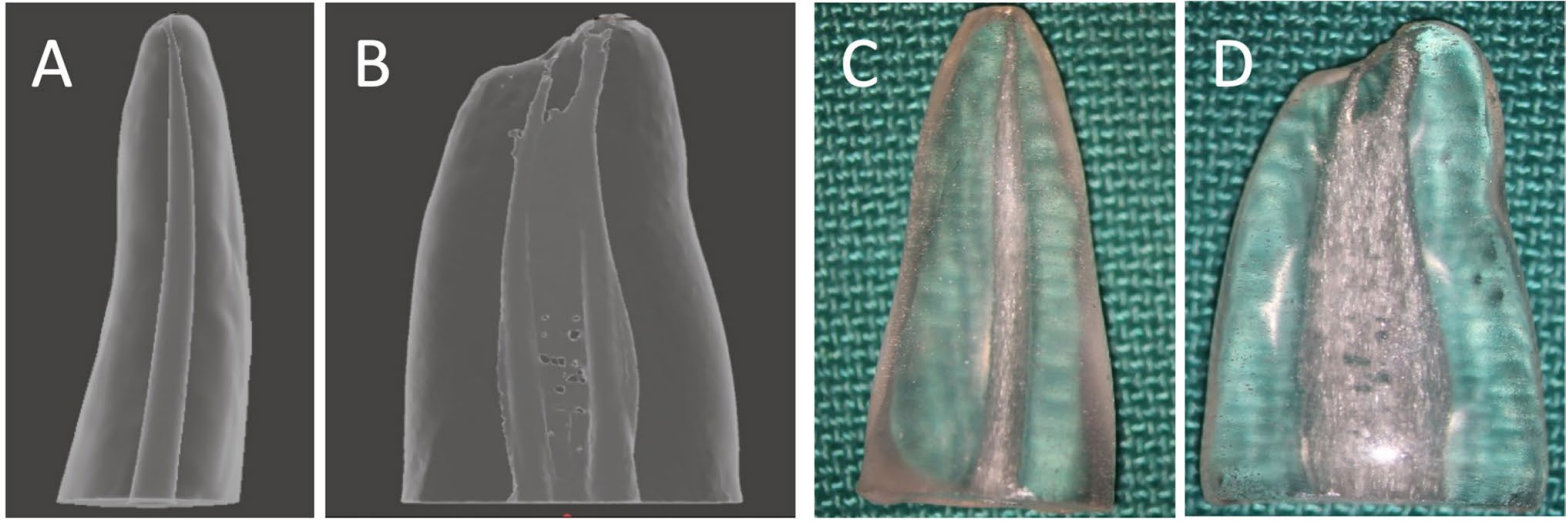
Digital and 3D printed acrylic replicas; **A** digital model of the incisor, **B** digital model of the molar, **C** printed incisor, and **D** printed molar. Note the similarity of the root canal morphology of polymer teeth compared with digital models

To make the 3D printed acrylic replicas, a number of upper incisors and lower molars were scanned using µCT (Bruker Skyscan MicroCT model 1172, Billerica, MA, USA) from apex to crown at 11 µm intervals, using an accelerating voltage of 70 kV at 140 mA and medium camera pixels (2 K × 1 K). These parameters were used for all µCT scans throughout the experiment. The scanned images were reconstructed using the NRecon software (Bruker, Billerica, MA, USA) and saved in .tiff file format. An incisor root with a straight circular continuously tapered canal from the coronal to the apical part of the root (Fig. 1A) was selected as a simple canal morphology tooth. A molar with a mesial root having two continuously tapered canals from the coronal to the apical part of the root, two straight separate apical foramina, and a continuous isthmus along the root length (Fig. 1B) was selected for complex root canal anatomy. 3D root canals and dentine images of these teeth were created using CTAn software (Bruker) and were saved in the .stl file format. The selected digital files were then printed in resin (RepliDens®, Smartodont, Zurich, Switzerland), thus providing one round uncomplicated canal and a complex root canal system for obturation (Fig. 1C and D).

### Root canal obturation

The prepared natural roots and the replicas of both the single-rooted teeth and the mesial roots of the lower molar were obturated using either a single cone/sealer-based technique or warm vertical compaction (WVC) with the test sealers. Master cones (ProTaper Gold) F3 and F2 were fitted into the incisors and molar roots respectively. An additional group was added where Totalfill BC sealer was used. Besides using the standard gutta-percha master cone, a bioceramic coated (BC) gutta-percha (Totalfill) was used in conjunction with the sealer-based technique. For WVC, the down packing stage was performed using a presized heat carrier (EQ-V, Meta Biomed, Chungcheongbuk-do, Republic of Korea) that precisely fitted in the canal at 3–5 mm from WL, while the back-filling stage was performed using thermoplasicized syringeable gutta-percha (EQ-V, Meta Biomed, Chungcheongbuk-do, Republic of Korea). The coronal orifices were restored with glass ionomer cement (ChemFil® Superior, Dentsply DeTrey, Konstanz, Germany). Five roots per technique and tooth type were used. After completion of obturation, all teeth were immersed in 1.5 mL of HBSS at 37 °C.

### Gap and void volume measurement

In order to establish the correct parameters to test the percentage of gaps and voids (%*V*_*gv*_), a preliminary study was undertaken to establish whether the experimental set-up influenced the data generated.

#### Preliminary study

A preliminary study was designed to determine how fluctuations in machine temperature, tooth positioning, and the moisture conditions of the specimens could have affected the µCT measurements. The experiments were carried out on human single-rooted teeth that were prepared as described above and also on the replicas of the incisor roots.

To test the effect of the µCT temperature on the readings, the machine was started and was kept on for 3 h. A thermocouple device (data logger model TC-08, Pico® technology, Saint Neots, UK) was used to measure the temperature inside the µCT machine by continuously recording it every second for 5 h. The µCT machine was operated in either an interrupted or continuous scan mode. For the interrupted scan mode, the X-ray beam was stopped every 53 min, rested for 1 min, and restarted with the method repeated five times to represent the actual working time for tooth scanning. A continuous scan mode was also employed where the X-ray beam was used continuously until the scan was completed. The initial, maximum, and average working temperatures were recorded by Picolog version 6.1.14 software (Pico® technology, Saint Neots, UK). The temperature of the machine chamber when operated in these modes was recorded. Five single-rooted of replicas were stored at the average operated machine temperature and were then scanned. Furthermore, these replicas were stored at room temperature, scanned, and then placed at 37 °C in an incubator until reaching a stable temperature and were rescanned. All teeth were scanned five times for each temperature and scanning mode used. All the scanned images were reconstructed using the NRecon software (ring artefact reduction = 13, beam-hardening correction = 20% and dynamic image range from 0.001 to 0.05) and saved in.tiff file format. All reconstructed images were imported into the Dataviewer software (Bruker) to 3D-register by overlapping two images from different scans together, and the root canal volume was then calculated.

To evaluate the effect of tooth positioning within the µCT chamber on the data generated, replicas were either fixed to a silicone putty that allowed reorientation after every scan or were just placed in the µCT machine in random positions. Each tooth was scanned using the same parameters as for the temperature assessment. All scanned images were reconstructed using the NRecon software. Tooth position was adjusted with a 3D-segmentation method, where the first scanned tooth image was imported as a reference, whilst another tooth image was imported as the target image. The target image was adjusted until it completely overlapped the reference image before exporting the adjusted image in a .tiff file format. The root canal volume of the initial position, altered position, and software-adjusted position were calculated using ImageJ.

To assess the effect of dehydration, both replicas and human teeth were used. These were scanned after storage in water and therefore fully saturated, storage at room temperature and also in a fully dehydrated state by storing in a desiccator with silica gel.

#### Measurement of %V_gv_ of test specimens

The teeth and replicas for both the incisors and molars were scanned pre- and post-obturation with µCT using the same parameters described for the scans of the teeth used to make the replicas. %*V*_*gv*_ were assessed with the CTAn software by determining the thresholding range for each type of sample which varied depending on the tooth type and the obturation method. The region of interest (ROI) was identified, following the outline of the gap and void area. The total %*V*_*gv*_ was calculated and compared between each group using these equations:

$$\%Vgv=\frac{\mathrm{gap}\;\mathrm{volume}+\mathrm{void}\;\mathrm{volume}}{\mathrm{canal}\;\mathrm{volume}}\times100$$

where $$\mathrm{canal}\;\mathrm{volume}=\mathrm{gap}\;\mathrm{volume}\;+\;\mathrm{void}\;\mathrm{volume}\;+\;\mathrm{filling}\;\mathrm{volume}$$.

The teeth were assessed immediately after obturation and again after a further 3 and 6 months. To simulate the clinical environment, a simulated periodontal ligament was painted on the natural roots. This consisted of a layer of 0.1–0.4 mm thickness of collagen-agarose gel containing 1 mg/mL of collagen (FibriCol®, Advanced BioMatrix, San Diego, CA, USA) and 1% w/v agarose (Bioline, London, UK) made up in HBSS (Sigma-Aldrich, Gillingham, UK) and the pH adjusted to 7.4. The teeth were then immersed in 1.5 mL HBSS and stored in an incubator at 37 °C for 3 or 6 months. The replicas were also immersed in HBSS but without any surface modification. After 3 and 6 months, the coating was gently removed using wet gauze; the teeth rescanned, reconstructed, and 3D-segmented using the same methods as before to enable calculation of %*V*_*gv*_. The tooth was recoated with collagen/agarose gel and re-immersed in HBSS in an incubator at 37 °C. The %*V*_*gv*_ data obtained immediately after obturation was compared with 3 and 6 months data.

### Leachate analysis

After 3 and 6 months, 1 mL HBSS were collected and diluted 10-fold and leachate analysis was performed to measure calcium ion leaching using inductively coupled plasma mass spectroscopy (Optima 8000; Perkin Elmer, Waltham, MA, USA).

### Statistical analysis

All statistics were performed using the SPSS software version 23 (IBM, North Castle, NY, USA). A Kolmogorov−Smirnov test was used to test the normality of the data. Intrarater reliability was calculated by using intraclass correlation coefficient. The Wilcoxon signed-rank test was used to test the effect of the temperature of the tooth on the precision of µCT. The Friedman test was used to test the effect of the moisture and the position of the tooth on the precision of µCT. The mean canal volumes between each group in the same tooth type were compared using one-way repeated analysis of variance (ANOVA) test and post hoc analysis. %*V*_*gv*_ at each period (post-obturation, 3 months, and 6 months) were compared between different sealers using the Kruskal−Wallis test and Dunn−Bonferroni analysis. %*V*_*gv*_ from the same sealer group at the different periods were compared between each sealer using the Friedman test and Dunn-Bonferroni analysis.

## Results

### Sealer characterisation

**Fig. 2:**
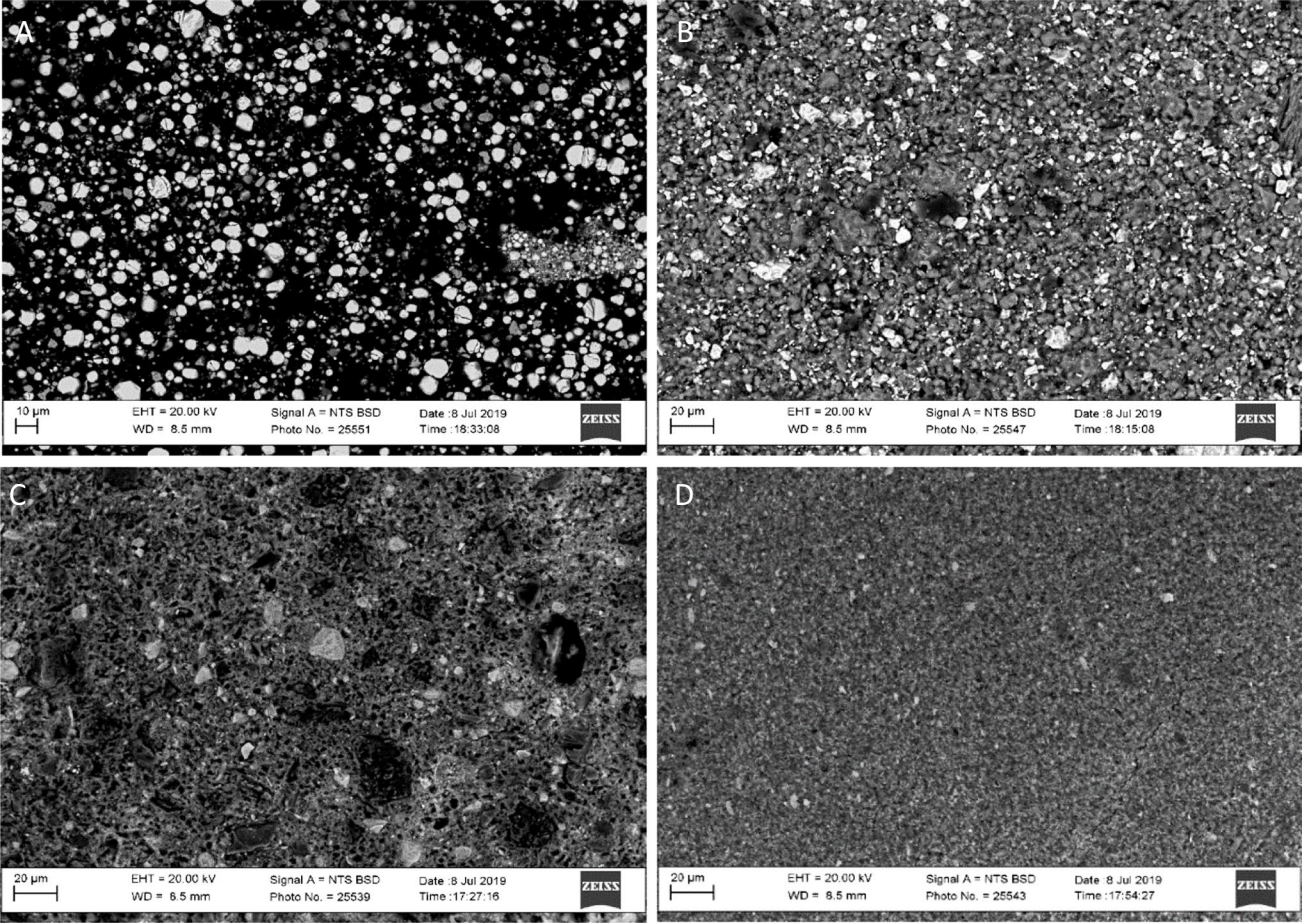
Back scattered scanning electron micrographs of the four test sealers showing their microstructure; **A** AH Plus, **B** BioRoot RCS, **C** Totalfill BC Sealer, and **D** Bio-C Sealer

The microstructure and the mean elemental composition of each sealer are shown in Fig. 2 and Table 1, respectively. AH Plus included discrete particles (Fig. 2A) composed of zirconium and tungsten embedded in a calcium-rich matrix (calcium tungstate) as indicated in Table 1. All HCSC sealers were composed of numerous calcium-rich particles with zirconium and silicate particles dispersed within the cement matrix (calcium silicate) (Fig. 2B–D). Particle size varied with AH Plus and BioRoot RCS having particles 2–10 µm. Totalfill BC particle size was approximately 4–15 µm and Bio-C Sealer appeared the smallest with an average particle size of 1 µm. Among all sealers, BioRoot RCS had the highest calcium level and AH Plus was the only sealer that contained tungsten (Table 1).

**Table 1:** Semi-quantitative elemental composition of the test sealers

Sealer	Element (mean % weight fraction)				
	Ca	Si	Zr	W	Al
AH Plus	2.5 ± 0.5	BDL	10.8 ± 0.5	12.7 ± 2.4	BDL
BioRoot RCS	23.8 ± 2.9	6.5 ± 0.7	17.0 ± 1.5	BDL	BDL
Totalfill BC	9.5 ± 0.8	2.9 ± 0.2	24.3 ± 2.0	BDL	BDL
Bio-C Sealer	4.3 ± 2.7	1.2 ± 0.6	17.3 ± 5.7	BDL	0.5 ± 0.2

### Gap and void volume measurement

#### Preliminary study

**Fig. 3:**
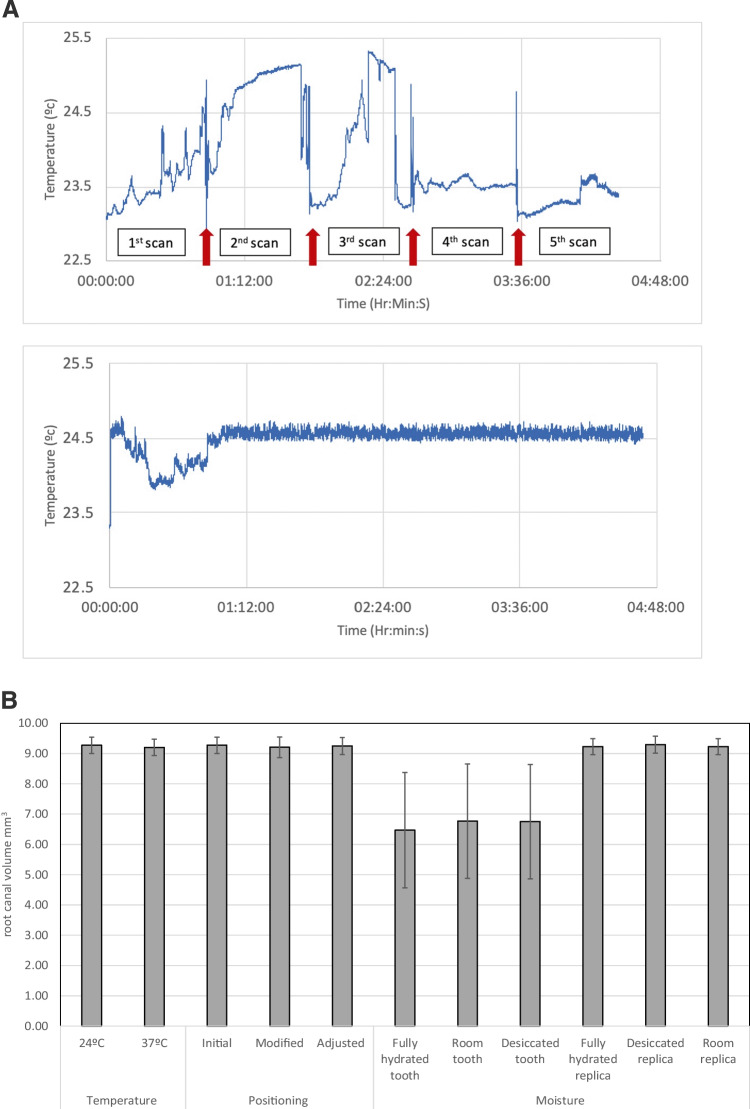
**a** The µCT working temperature readings from a non-continuous scan. Red arrows show the temperature when the µCT chamber was opened and a continuous scan. The temperature was stable approximately 50 min after initiated the µCT scan. **b** Changes in root canal volume as a function of temperature, positioning, and moisture condition of the tooth

In the µCT continuous working mode, it took an hour for the temperature to stabilize, while in the non-continuous working mode, there were temperature fluctuations with each scan (Fig. 3a). There was no significant difference in root canal volume measured at different temperatures, moisture, or position within the scanner (*p* > 0.05) as shown in Fig. 3b.

#### Measurement of gap and void volume of test specimens

**Fig. 4:**
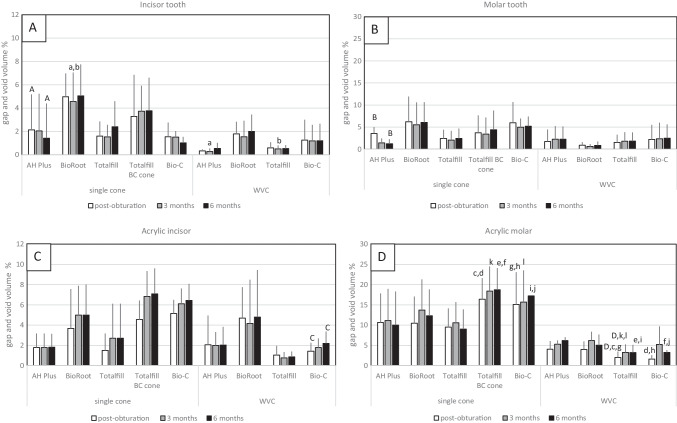
Mean percentage of gaps and voids of natural and 3D printed replica teeth from post-obturation, 3 months and 6 months; **A** natural incisor group, **B** natural molar group, **C** 3D replica incisor group, and **D** 3D replica molar group (*n* = 5) (mean ± SD). (A, B, C, and D) Same letter means a significant difference (*p* ≤ 0.05) between each period within the same sealer type and technique. (a, b, c, d, e, f, g, h, i, j, k, and l) Same letter means a significant difference (*p* ≤ 0.05) between each sealer type and obturation technique at the same period

%*V*_*gv*_ for each tooth type in both natural teeth and 3D printed acrylic replicas is shown in Fig. 4. The mean empty root canal volume was the same for all teeth in a specific tooth type and also did not vary between the natural teeth and the 3D printed acrylic replicas (*p* > 0.05). The %*V*_*gv*_ of obturated human teeth was the same as that of the replicas in the incisor group (*p* = 0.159) but not in the molars (*p* < 0.001) indicating that acrylic replicas could be used to investigate obturation quality in teeth with simple canal anatomy but not in molar teeth with more complex anatomy.

The quality of obturation in natural teeth was not dependent on the technique used (*p* > 0.05) but was affected by the type of sealer. In both incisors and molar teeth obturated with a single cone and AH Plus sealer, the %*V*_*gv*_ at 6 months were significantly decreased compared with post-obturation (Fig. 4A, superscript A, and Fig. 4B, superscript B; *p* < 0.05). All the other sealers and techniques exhibited no significant changes over the 6-month period. At 3 months, WVC obturations with AH Plus and Totalfill BC had less voids than were identified with the BioRoot RCS single cone/sealer-based technique (Fig. 4A, superscripts a and b; *p* < 0.05).

In the 3D printed acrylic incisors, the obturation with Bio-C Sealer and WVC deteriorated significantly over the 6-month period (Fig. 4C, superscript C; *p* < 0.05). In the 3D printed acrylic molars, the Totalfill BC WVC obturation deteriorated at 3 months compared with post-obturation (Fig. 4D, superscript D; *p* < 0.05). The higher sealer levels for the premixed sealers (Bio-C Sealer and Totalfill BC Sealer) used in single-cone obturation technique led to more gaps and voids at 6 months (Fig. 4D, superscripts c-j; *p* < 0.05) and 3 months (Fig. 4D, superscript k and l; *p* < 0.05) compared with the same sealers used in WVC.

### Leachate analysis

The leachate data is shown in Table 2 where BioRoot RCS exhibited the highest release of calcium. The leaching was higher in the single cone/sealer-based technique compared with the WVC in natural teeth (*p* < 0.05). This decreased after 6 months for all HCSC sealers in both natural teeth and replicas except for BioRoot RCS in the natural teeth where a higher calcium ion release was noted. Bio-C sealer released the lowest amount of calcium ions at each time point. The Totalfill BC sealer used with the BC cone had a higher calcium ion leaching than when used with a standard cone in the incisor tooth and not in molars and replicas (*p* < 0.05). AH Plus released the least amount of calcium ions in every tooth type (Table 2). This increased after 6 months in the natural teeth and was reduced in the replicas.

**Table 2:** Calcium ion concentration in ppm leached from each tooth or replica using different obturation techniques and different sealers

Tooth type	Obturation technique	AH Plus		BioRoot RCS		Totalfill BC		Totalfill BC cone		Bio-C Sealer	
		3 months	6 months	3 months	6 months	3 months	6 months	3 months	6 months	3 months	6 months
Natural incisor	Single cone	0.0	8.3 ± 3.6	40.3 ± 1.7	99.0 ± 8.1	88.5 ± 6.6	33.2 ± 5.8	231.2 ± 3.3	12.7 ± 13.8	16.7 ± 5.6	5.6 ± 4.1
	WVC	0.0	12.2 ± 9.2	19.8 ± 1.9	46.7 ± 6.3	7.1 ± 9.8	9.0 ± 11.5	/	/	7.6 ± 7.5	2.7 ± 2.6
Natural molar	Single cone	1.0 ± 0.2	11.4 ± 1.3	0.8 ± 0.7	159.0 ± 5.3	6.1 ±7.8	82.8 ± 27.7	8.2 ± 2.4	44.7 ± 4.9	13.2 ± 8.5	15.9 ± 15.3
	WVC	1.4 ± 0.2	14.9 ± 5.4	1.1 ± 0.9	99.8 ± 26.1	28.7 ± 9.5	24.4 ± 9.9	/	/	0.3 ± 0.03	7.9 ± 4.8
Replica incisor	Single cone	66.5 ± 0.7	7.5 ± 0.2	99.3 ± 6.0	5.8 ± 2.1	87.2 ± 6.2	38.4 ± 2.1	86.8 ± 6.3	14.5 ± 3.6	79.9 ± 19.5	22.9 ± 12.3
	WVC	56.0 ± 7.2	17.0 ± 8.7	104.4 ± 6.4	21.2 ± 10.8	77.4 ± 16.2	30.1 ± 2.2	/	/	85.2 ± 1.1	13.1 ± 6.3
Replica molar	Single cone	62.5 ± 4.1	8.6 ± 1.5	250.8 ± 7.8	6.2 ± 0.6	138.8 ± 7.9	6.9 ± 0.5	113.8 ± 22.35	6.1 ± 2.8	78.9 ± 6.5	11.3 ± 10.9
	WVC	67.4 ± 7.2	5.7 ± 1.9	225.6 ± 5.4	5.9 ± 1.2	94.5 ± 13.8	4.6 ± 1.2	/	/	75.1 ± 2.5	47.5 ± 3.0

## Discussion

The current study investigated the assessment of quality of obturation using microcomputed tomography to measure %*V*_*gv*_ of complex and simple root canal systems obturated using either WVC or single-cone/sealer-based techniques and four different sealers. The sealers investigated in the current study included AH Plus, an epoxy-resin-based sealer which was used as the control as it is widely used and well established. The test sealers included a water-based hydraulic cement sealer (BioRoot RCS), and two syringeable hydraulic cement sealers that were formulated using a non-aqueous vehicle. These materials were selected to assess their interaction with the dentine substrate and whether it was possible to eliminate the voids and gaps within the obturation. All the sealers used in the study were characterized to establish their chemistry and microstructure. Material characterization is key to be able to predict and adequately assess the clinical behaviour of the sealers under investigation.

The advantage of measuring %*V*_*gv*_ using µCT allows longitudinal testing of the obturation as the technique is non-invasive unlike other techniques [17] that have been employed to study leakage. In the present study, both natural teeth and replicas were used as natural teeth are inherently variable [18–23] making the acquired data difficult to analyse and interpret. The measurement of %*V*_*gv*_ of replicas generated similar data as that from natural teeth in the incisor group but not for the molars. This indicated the suitability of replicas for assessment of quality of obturation in uncomplicated root canal systems only. The use of replicas is a convenient way to replace natural teeth as each have the same root canal morphology making it easier to compare the data obtained for the %*V*_*gv*_ assessment.

The objectives of obturation materials are to serve as a barrier to prevent the communication between oral cavity and periapical area and to entomb any remaining microorganisms within the root canal [24]. To date, several HCSC sealers have been introduced which have been claimed to be stable and do not expand when set [25, 26] but also have bactericidal properties [5–8]. These materials are thus suggested for use in sealer-based obturation techniques with a single gutta-percha point that is used as a pilot to facilitate removal if necessary. However, the use of high volumes of sealer although having bactericidal properties, in single matched cone obturation, may have the disadvantage of less canal space being filled as less effort is made to compress and manipulate the obturation material into the complex root canal systems. In the current study, single-rooted teeth and also mesial roots of lower molars were used to assess whether the complexity of root canal system influenced the obturation quality. There was no significant difference in %*V*_*gv*_ between simple root canals and complex root canals, which indicated the complexity of root canal system was not a factor influencing obturation quality.

Hydraulic cements release calcium ions which in turn interact with the clinical environment. For sealers, the interaction is with the root dentine [27] and the sealer chemistry has been shown to affect the antimicrobial properties [7]. EDS showed that all hydraulic cement sealers were composed primarily of calcium and silicon which is in agreement with previous studies [28–31]. The use of replicas can potentially impede penetration of environmental moisture into the root canal thus effecting the interaction of the sealers. The heat from WVC may also cause evaporation of moisture within the root canal [32]. The highest calcium ion content identified using EDS was found in BioRoot RCS followed by Totalfill BC and Bio-C Sealer, respectively. This reflected the leachate data that identified BioRoot RCS, and Bio-C Sealer released the highest and the lowest amount of calcium ions, respectively. The leaching was higher in sealer-based obturation compared with the WVC in natural teeth. The replicas showed lower calcium ion leaching than was measured from natural teeth. This may be explained by the interference of the hydration reaction that the sealer would have with the dentine substrate. In this respect, although no variation was found in %*V*_*gv*_ when using resin replicas, clearly, the sealer hydration and interaction with dentine were affected. The leaching occurred despite no significant variation in the %*V*_*gv*_ clearly showing the distinction between the solubility which is a physical process off material loss and ion leaching which is a chemical process.

The use of warm vertical compaction aims at having an obturation that is composed primarily of gutta-percha. The use of heat can negatively affect the sealer properties. AH Plus can set faster and decompose when heated excessively [32–34] although it has been shown that the temperature inside the root canal does not rise to more than 60 °C [35] and AH Plus will only deteriorate if heated indiscriminately [36]. In the current study, AH Plus and Totalfill BC sealers performed well when used with warm vertical obturating technique. Thee Totalfill BC sealer has been shown to be heat resistant [37], and this could account for the low gap and void volume shown in this study.

High %*V*_*gv*_ was identified in most teeth when using BioRoot RCS, which may be related to its high viscosity and manual mixing, where air could be entrapped inside the material [38]; therefore, the material could contain voids even before the obturation.

Another sealer property that could contribute to higher void volumes is the solubility. Although solubility has been reported to be high for HCSC sealers [39, 40], this was sown to depend on the environment the sealers were placed in. Thus, solubility was shown to be reduced when in contact with simulated tissue fluids [41, 42]. The AH Plus was not susceptible to the solubility, and it exhibited no voids after 6 months immersion.

High void percentage was also found in the BC cone group. BC cones have a taper of 4% which was smaller than both the ProTaper Gold F2 and F3 (various taper of 8% and 9%, respectively). Hence, the amount of gutta-percha was insufficient to spread the sealer into voids. This finding contrasted with the sealer-based concept, where the gutta-percha is used as a carrier and a path for retreatment only.

## Conclusions

HCSC sealers could be used to obturate both complex and simple root canals using either WVC or single cone/sealer-based techniques. BioRoot RCS with sealer-based technique should be used when calcium ion release is clinically useful. 3D printed acrylic teeth can be used to assess the obturation quality in uncomplicated root canal systems. Temperature, position, and moisture of the tooth had no significant effect on the µCT measurement.

## Data Availability

The data sets can be accessed on request to corresponding author.
